# Single Fringe Phase Retrieval for Translucent Object Measurements Using a Deep Convolutional Generative Adversarial Network

**DOI:** 10.3390/s25061823

**Published:** 2025-03-14

**Authors:** Jiayan He, Yuanchang Huang, Juhao Wu, Yadong Tang, Wenlong Wang

**Affiliations:** 1 School of Mechanical and Electrical Engineering, Guangzhou University, Guangzhou 510006, China; 2112207008@e.gzhu.edu.cn (J.H.);; 2 School of Biomedical and Pharmaceutical Sciences, Guangdong University of Technology, Guangzhou 510006, China

**Keywords:** fringe projection profilometry, phase retrieval, generative adversarial network, translucent objects

## Abstract

**Highlights:**

**What are the main findings?**

- An improved GAN network is proposed, capable of obtaining wrapped phase information using a single image.
- A method is introduced for obtaining high-precision phase information of translucent objects affected by scattering effects.

**What is the implication of the main finding?**

- The proposed method demonstrates greater robustness compared with traditional FPP algorithms and conventional deep learning-based methods.
- It retrieves more accurate phase information when the object’s surface is significantly affected by scattering effects, enabling better analysis of complex translucent objects.

**Abstract:**

Fringe projection profilometry (FPP) is a measurement technique widely used in the field of 3D reconstruction. However, it faces issues of phase shift and reduced fringe modulation depth when measuring translucent objects, leading to decreased measurement accuracy. To reduce the impact of surface scattering effects on the wrapped phase during actual measurement, we propose a single-frame phase retrieval method named GAN-PhaseNet to improve the subsequent measurement accuracy for translucent objects. The network primarily relies on a generative adversarial network framework, with significant enhancements implemented in the generator network, including integrating the U-net++ architecture, Resnet101 as the backbone network for feature extraction, and a multilevel attention module for fully utilizing the high-level features of the source image. The results of the ablation and comparison experiment show that the proposed method has superior phase retrieval results, not only achieving the accuracy of the conventional method on objects with no scattering effect and a slight scattering effect but also obtaining the lowest errors on objects with severe scattering effects when compared with other phase retrieval convolution neural networks (CDLP, Unet-Phase, and DCFPP). Under varying noise levels and fringe frequencies, the proposed method demonstrates excellent robustness and generalization capabilities. It can be applied to computational imaging techniques in the fringe projection field, introducing new ideas for the measurement of translucent objects.

## 1. Introduction

The fringe projection profilometry (FPP) technique [1], as a form of structured light measurement technology, is extensively employed across various domains, including industrial inspection [2], biomedical applications [3], digital heritage preservation, and entertainment [4,5], attributed to its non-contact modality, superior reconstruction accuracy, expeditiousness, and cost-effectiveness. The FPP system is conventionally composed of the following three primary components: a projector, a camera, and a computer. By projecting sinusoidal fringe patterns onto an object, the object’s height information is encoded into the phase of the pattern, leading to the modulation of patterns. The captured fringe images are processed by a fringe analysis algorithm that extracts the phase distribution and recreates the surface of interest in three dimensions based on the geometrical relationships of the triangulation optical arrangement.

However, traditional FPP techniques often encounter difficulties when measuring translucent objects, including marble, teeth, candles, and industrial components made from plastic resin, primarily due to subsurface scattering effects [6]. This challenge stems from two main factors. First, depth displacement occurs as light from the source illuminating the object, with some penetrating its interior and undergoing multiple reflections before reaching the sensor. This process results in inaccuracies, as some light originates from within the object rather than its surface [7]. Second, image contrast degradation occurs when patterns are projected onto translucent surfaces. Subsurface scattering reduces the modulation of the fringe patterns, leading to blurred images captured by the camera. This results in incomplete measurements and substantial errors, ultimately diminishing reconstruction accuracy [8].

Measurement methods for translucent objects have been extensively studied. Chen et al. [9] placed a polarizer to reduce the subsurface scattering. Nayar et al. [10] advanced the technique by isolating direct and global components from images illuminated at high frequencies, effectively eliminating scattering effects. Furthermore, Chen et al. [11] integrated this separation principle into a phase-shift method with modulated high frequencies, facilitating the measurement of translucent objects. Gupta et al. [12] suggested restricting the phase-shifted fringe pattern to a narrow high-frequency band to minimize geometric errors in the measurements. Additionally, Kobayashi et al. [7] introduced an approach that projected multiple sets of sparse dashed lines to eliminate subsurface scattering, achieving high-accuracy measurements. Despite these advancements, these methods remain reliant on traditional frameworks, necessitating the projection of multiple patterns for effective measurement. However, when the optical system is under severe measurement conditions or the state of the object is dynamically changing, the method of projecting multiple patterns would be severely limited in providing accurate phase information. Therefore, a way to achieve the most precise phase measurement possible from the minimum number of (preferably single) fringe patterns remains one of the most challenging problems in the FPP method [9].

In recent years, the advent of artificial intelligence has revolutionized optical measurement techniques, with deep learning becoming increasingly prevalent. Researchers have utilized deep learning to extract phase information from a single pattern, enabling high-precision measurements [10,11]. Feng et al. [12] were pioneers in applying deep learning to streak analysis, integrating this technology into Fourier transform profilometry (FTP) measurements. Zhang et al. [13] combined the phase-shift principle with deep learning, allowing three-step phase shifting to be input into a network that outputs the numerator and denominator (N and D) of the inverse tangent function, producing wrapped phase information. Qian et al. [14] employed two parallel U-Net architectures to achieve alias-free phase recovery from dual-frequency composite fringe maps, circumventing the complexities of dual-frequency spectral separation and extraction inherent in conventional spatial frequency-multiplexed FT methods. Yin et al. [9] introduced a learning-enhanced Fourier transform profilometry (LeFTP) module within a physical deep learning approach (PI-FPA) for fringe analysis, embedding a priori physical information into the network structure and loss function to directly provide reliable phase information. This utilization of deep learning has allowed researchers to overcome the limitations of traditional methods, such as reducing the number of images required for multi-frame measurements, thereby advancing high-precision single-frame FPP algorithms. While deep learning has significantly advanced FPP measurements, several challenges remain. First, the majority of the studies above have been conducted on plaster objects, with limited investigations focused on the surface measurements of translucent materials. Second, the majority of these studies rely on Convolutional Neural Networks (CNNs), with few exploring Generative Adversarial Networks (GANs). As a powerful generative model, a GAN can improve the detail accuracy and realism of images through adversarial training [15]. Therefore, for the measurement of translucent objects, the GAN is projected to leverage its characteristics to mitigate phase errors and information loss, which traditional methods have faced during the acquisition of phase data. Following this, the GAN is expected to facilitate the restoration and correction of the wrapped phase.

To this end, we propose a method for phase retrieval based on multilevel feature fusion, called GAN-PhaseNet, utilizing a Generative Adversarial Network (GAN) and treating fringe decoding as an image transformation task. This network is primarily designed for translucent objects that are affected by scattering effects, with the goal of restoring high-precision wrapped phase information that has been impacted by scattering. Subsequently, it combines this restored phase information with the corresponding fringe order map to derive the continuous phase information of the target object. Experimental results demonstrate that our algorithm achieves superior measurement accuracy compared to conventional FPP algorithms, mainly when dealing with translucent objects affected by significant scattering effects. The principal contributions of this work are as follows:

- The proposed GAN-PhaseNet enables the accurate acquisition of wrapped phase information using only a single fringe image, offering a novel approach for the surface measurement of translucent objects.
- We integrate the U-net++ architecture, Resnet101 as the backbone network for feature extraction, and a multilevel attention module for fully utilizing the source image’s high-level features, aiming to boost the generator’s feature extraction capability and generation accuracy.
- To train the GAN-PhaseNet model, we create a new dataset using our constructed FPP experimental system. This dataset focuses on translucent objects and comprises 1200 image groups, each including a fringe pattern, the numerator, and the denominator of the inverse tangent function. This dataset ensures the authenticity of the data by excluding any simulation-generated maps and providing high-quality, accurate, and reliable data to support model training.

The structure of the paper is as follows: Section 2 details the principles and the proposed network architecture. Section 3 presents the experimental results and discusses these findings. Section 4 outlines future research directions.

## 2. Materials and Methods

### 2.1. Principle of FPP

**Figure 1:**
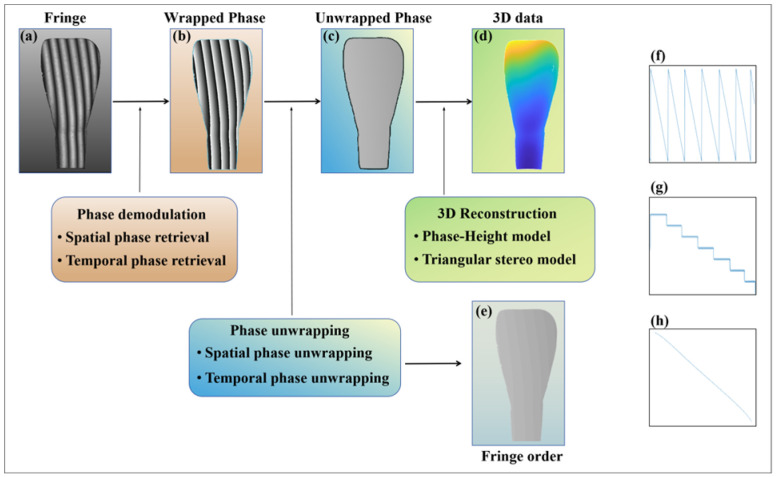
Measurement process of FPP algorithm. (**a**) Modulated fringe image; (**b**) Wrapped phase map; (**c**) Absolute phase map; (**d**) 3D point cloud map; (**e**) Fringe order map; (**f**), (**g**) and (**h**) are the pixel value waveforms of the 200th row in figures (**b**), (**e**) and (**c**) respectively.

The complete measurement process of the FPP technique is illustrated in Figure 1. The captured deformed fringe pattern is shown in Figure 1a and can be represented as Equation (1), as follows [16]:

(1) I(x,y)=A(x,y)+B(x,y)cos[ϕ(x,y)]

where A denotes the background intensity, B denotes the fringe amplitude, ϕ is the phase of the object under test, and (x,y) represents the camera pixel coordinate. In order to extract the object’s information from the captured camera image, accurate phase retrieval, and phase unwrapping are essential processes. Phase retrieval is performed to obtain the wrapped phase map, as shown in Figure 1b. The retrieval methods can be categorized into single-frame and multi-frame fringe pattern retrieval. Single-frame retrieval methods include Fourier transform profilometry (FTP) [17], windowed Fourier transform (WFT) [18], and wavelet transform (WT) [19]. However, when the measurement object exhibits sharp edges or discontinuities, the single-frame measurement method is susceptible to spectral overlapping, resulting in measurement errors and complicating the attainment of high-precision reconstruction results in practical measurements. Multi-frame pattern retrieval, on the other hand, can acquire more phase information, thereby facilitating the attainment of high-precision reconstruction results. The most widely used method is phase-shifting profilometry (PSP), and the N-step phase-shifting fringe patterns can be represented as follows [20]:

(2) $$I_{n}(x,y)=A(x,y)+B(x,y)\cos[\phi (x,y)-2\pi n/N],n\in [1,N],$$

where $I_{n}$ is the nth fringe image, N is the number of periods of the fringe patterns, and the following formula can calculate the wrapped phase:

(3) $$\phi (x,y)=\tan^{-1}\frac{\sum_{n=0}^{N-1}I_{n}(x,y)\sin[2\pi n/N]}{\sum_{n=0}^{N-1}I_{n}(x,y)\cos[2\pi n/N]}=\tan^{-1}\frac{M}{D},$$

where M and D represent the numerator and denominator of the arctangent function. Extracting a line of pixel values from the wrapped phase map results in a waveform, as illustrated in Figure 1f. Since the wrapped phase does not establish a direct, pixel-unique correspondence between the camera and the projector, an unwrapped phase must be executed to derive a continuous phase distribution, as shown in Figure 1c. The absolute phase can be obtained by the following formula [21]:

(4) ψ(x,y)=ϕ(x,y)+2πk(x,y),

where k(x,y) is the fringe order of the point (x,y), which is the most critical factor in the phase unwrapping process, as depicted in Figure 1e. The distribution of the fringe order and the absolute phase for a line of pixel values is illustrated in Figure 1g and Figure 1h, respectively. Finally, based on the calibration parameters of the system, the 3D shape of the object can be derived.

### 2.2. Network Architecture

**Figure 2:**
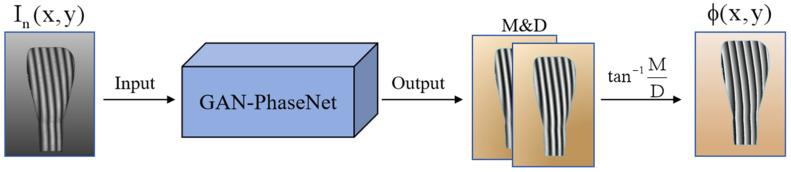
Flow of measuring objects.

When using a phase shifting pattern to perform the 3D measurement of translucent objects, the subsurface scattering effects lead to phase error and modulation degradation within the modulated fringe patterns, thereby yielding low-precision wrapped phases. To address this problem, the present study proposes the GAN-PhaseNet model, which is designed to achieve high-precision surface measurements and phase information extraction. The processing flow of the GAN-PhaseNet model is meticulously depicted in Figure 2. It is precisely engineered to generate two distinct images, designated as M and D, each representing a specific physical aspect. Subsequently, employing Equation (3), the wrapped phase can be computed using M and D. This process is realized through the utilization of a single input image, which not only streamlines the measurement process but also enhances the phase accuracy of translucent objects compared to the conventional method PSP.

**Figure 3:**
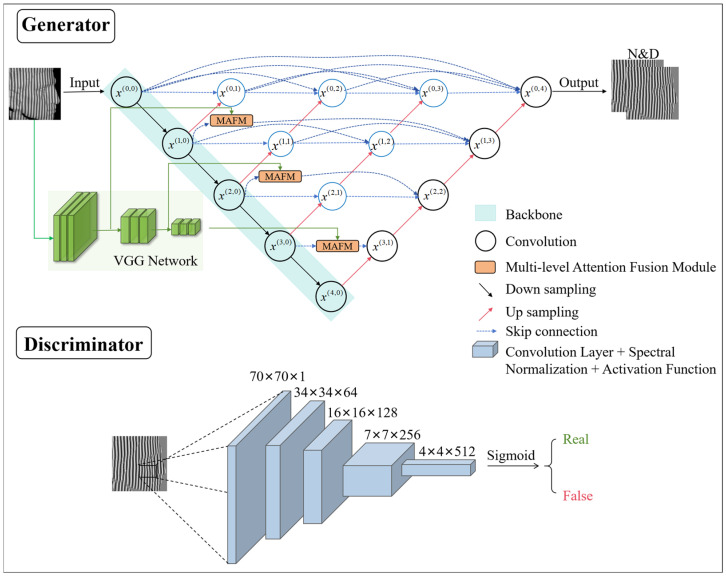
GAN-PhaseNet network architecture.

The core of the proposed GAN-PhaseNet model lies in the utilization of Generative Adversarial Networks (GANs). GANs are powerful generative models that have demonstrated their effectiveness in generating high-quality, high-precision images through adversarial training [22,23,24]. Within this adversarial framework, GANs’ generators and discriminators iteratively enhance the generator’s output to create images indistinguishable from real ones, significantly improving image quality and detail. By incorporating GANs, the GAN-PhaseNet model leverages this adversarial training process to enhance the quality of the generated wrapped phases, considerably improving the accuracy of phase-shift measurements. As shown in Figure 3, the main improvement of the proposed model is in the generator. Its operational mechanism closely parallels that of pix2pix [22], both learning through labeling processes. The generator enhancements encompass the following:

1. Adopting the U-net++ structure as the core architecture of the generator and adding multilevel attention fusion modules at the longest jump connections of levels 2, 3, and 4. Additionally, a pre-trained VGG-19 is used as the second branch for source image feature extraction, whose feature maps primarily serve the fusion in multilevel attention fusion modules to enhance the network’s ability to capture multi-scale features.
2. Incorporation of the Resnet101 network into the backbone for feature extraction, aiding in capturing rich and hierarchical features from input images to enhance overall feature representation.
3. Integration of a multilevel attention module to improve the multi-scale expression ability of the network and increase the receptive field of the network to strengthen the connection between high-level and low-level feature maps.
4. An attention mechanism module was added to extract the local features of the focal region.

#### 2.2.1. U-Net++ Architecture

The U-net++ [23], a sophisticated evolution of the original U-net model, is proposed to nest and densely connect decoders at both the same and different scales via convolutional layers and skip connections. The incorporation of convolutional operations is applied to generate mid-level features, thereby alleviating the feature gap issue caused by the simple and direct skip connections in traditional U-net architectures. Building upon existing studies [11] that highlight the U-net architecture’s efficacy in obtaining fringe patterns, it is hypothesized that the U-net++ architecture can yield superior fringe patterns.

The overall structure of the U-net++ is illustrated in Figure 3. Let $X^{\left(i,j\right)}$ denote the output of node $x^{\left(i,j\right)}$ where i indexes the down-sampling layer along the encoder and j indexes the convolution layer of the dense block along the skip connection. When i and j are zero, $x^{\left(0,0\right)}$ represents the network input. The images at the node $x^{\left(0,4\right)}$ are processed through a 1 × 1 convolution kernel to integrate the multi-channel images back into a single-channel image as the network output. In our current work, the nodes $x^{\left(0,0\right)}$, $x^{\left(1,0\right)}$, $x^{\left(2,0\right)}$, $x^{\left(3,0\right)}$, and $x^{\left(4,0\right)}$ represent images of size 512 × 512, 256 × 256, 128 × 128, 64 × 64, and 32 × 32, respectively, with the number of channels being 64, 128, 256, 512, and 1024. All of this information is determined by the backbone’s adoption of the Resnet101 network. Based on the original U-net++ architecture, the following improvements were made: as shown in Figure 3, a multilevel attention fusion module was added at the longest connections in levels 2, 3, and 4. This module incorporates a pre-trained VGG-19 network for feature extraction, enhancing the U-net++ network’s multi-scale representation capabilities, expanding the receptive field, and strengthening the connections between high-level and low-level feature maps. Detailed information about the multilevel attention fusion module is provided in Section 2.2.3.

#### 2.2.2. Backbone Extraction Network

**Figure 4:**
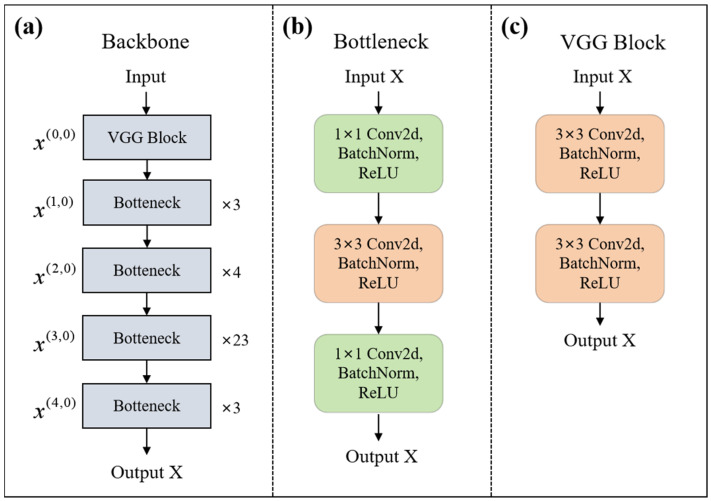
Architecture and details of the backbone. (**a**) Structural composition of the backbone network; (**b**) Main composition of the bottleneck in (**a**); (**c**) Main composition of the VGG block in (**a**).

In order to improve the multi-scale expression of the network and strengthen the connection between high-level and low-level feature maps, we incorporated the Resnet101 network as the backbone for perceiving information at different scales, improving the multi-scale expression ability of the network and increasing the receptive field of each network layer. Figure 4a shows the details of the backbone. The bottleneck uses 1 × 1, 3 × 3, and 1 × 1 convolutions to map features, each convolutional layer followed by batch normalization and a ReLU activation function, as shown in Figure 4b. In the backbone network, the original 7 × 7 convolutional kernel is replaced with the VGG block to enhance computational efficiency and facilitate the more detailed extraction of local features. As shown in Figure 4c, a VGG block comprises two 3 × 3 convolutional kernels, each convolutional layer followed by batch normalization and a ReLU activation function.

#### 2.2.3. Multilevel Attention Fusion Module

Inspired by the work of J. Liu et al. [24], to tackle low-intensity or local information loss in the input fringe image due to scattering effects from the test object, a multilevel attention fusion module (MAFM) is employed. This module facilitates the network in extracting more sophisticated features from the source image, thereby achieving a comprehensive feature representation of the source image. As shown in Figure 3, the MAFM has been integrated into the longest jump-connected layer in the $x^{1}$, $x^{2}$, and $x^{3}$ layers of the network. Furthermore, as feature map information may be lost during the long skip connection process, the inclusion of this module helps mitigate such losses, ensuring the better preservation of feature information.

**Figure 5:**
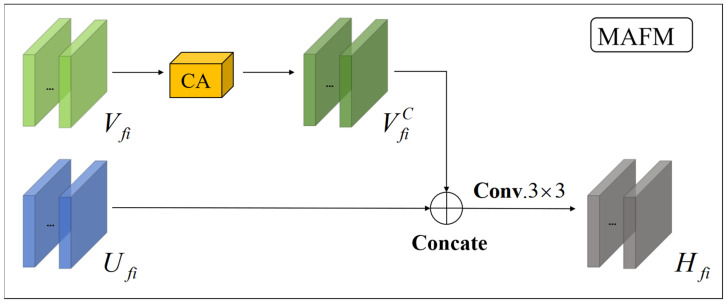
Internal schematic of the multilevel attention fusion module.

The specifics of the module are illustrated in Figure 5. This module integrates features from different levels to enhance the network’s ability to capture both global and local information. More specifically, it combines features extracted from the backbone network (ResNet101) with those from an additional feature extraction path using a pre-trained VGG-19 network. In the first branch, the pre-trained VGG-19 network separately takes the input image as an attempt to make full use of the high-level features of the source image. The feature maps extracted from this branch can be represented as $V_{fi}$, i∈[1,3]. In the second branch, feature maps are extracted from the nodes $x^{\left(1,0\right)}$, $x^{\left(2,0\right)}$, and $x^{\left(3,0\right)}$ of the Resnet101 network, and they can be represented as $U_{fi}$, i∈[1,3], i∈[1,3].

Additionally, in order to enhance the expressiveness of the features and suppress unimportant features while paying attention to the essential features, we select the channel attention mechanism (CA), added into the first branch, as shown in Figure 5. This mechanism dynamically adjusts feature channel weights to prioritize task-relevant features, significantly enhancing model performance. Its lower computational complexity and fewer parameters enable its practical deployment. Subsequent experimental results indicate a reduction in test error after incorporating channel attention. More specifically, the feature image $V_{fi}$ is transformed into ${V_{fi}}^{C}$ following the application of CA, and the output $H_{fi}$ of the MAFM can be acquired by convolving ${V_{fi}}^{C}$ and $U_{fi}$, where, i∈[1,3].

#### 2.2.4. Discriminator Architecture

Finally, a 70 × 70 PatchGAN discriminator [22] is employed, which is used for the classification of authenticity in overlapping 70 × 70 pixel image patches. The PatchGAN discriminator is a local patch-level classifier that offers advantages such as high computational efficiency and a smaller number of parameters compared to traditional full-image discriminators. It can be flexibly applied to images of arbitrary size in a fully convolutional manner. The core of the PatchGAN discriminator consists of multiple convolutional layers, each followed by spectral normalization and an activation function. Its specific structure is illustrated in Figure 3.

### 2.3. Loss Function

In the realm of GANs, choosing the appropriate loss functions plays a pivotal role in achieving stable training and optimal performance. In our proposed method, additional labeling information is fed into both the generator and discriminator to enable the model to guide the data generation process. So, the objective function for the training process is performed as follows [25,26]:

(5) $$L_{GAN}(G,D)={Ε}_{y_{0},y_{t}}[\log(D(y_{0},y_{t}))]+{Ε}_{y_{0},z}[\log(1-D(y_{0},G(y_{0},z))],$$

where G denotes the generator, D denotes the discriminator, $y_{0}$ is the original data, $y_{t}$ represents the labeled data, z is the random noise, and E is the mean operator. However, merely utilizing the objective Function (5) is insufficient for generating high-precision phase maps. Inspired by the work of P. Isola et al. [22], we incorporated both $L_{1}$ loss and $L_{MSE}$ loss to enhance the model’s performance, and these are defined as follows:

(6) $$L_{1}(G)={Ε}_{y_{0},y_{t}}[{\left\|y_{t}-G(y_{0})\right\|}_{1}],$$

(7) $$L_{MSE}(G)=E_{y_{0},y_{t}}[{\left(y_{t}-G(y_{0})\right)}^{2}],$$

The $L_{1}$ loss function, also known as the Mean Absolute Error (MAE), effectively mitigates the occurrence of image blur and ensures that the images in the source and target domains remain as close as possible. It demonstrates a lower sensitivity to outliers, making it particularly effective in scenarios where data may include numerous outliers, as it ensures these outliers do not disproportionately influence model performance. However, in our task, input images frequently suffer from excessive local information loss due to scattering effects, resulting in a high number of outliers. Thus, employing the $L_{1}$ loss function solely appears disadvantageous for these samples. Consequently, to further enhance network performance and stability, we incorporated $L_{MSE}$ loss into our loss function. The $L_{MSE}$ loss, in contrast, is more sensitive to outliers, implying that the model will focus more on inaccurate samples during training, thereby improving overall prediction accuracy [27]. Therefore, the training loss of the generation process is defined as follows:

(8) $$G^{*}=\arg\underset{G}{\min}\;\underset{D}{\max}\;L_{GAN}(G,D)+\lambda_{1}L_{1}(G)+\lambda_{2}L_{MSE}(G),$$

Through extensive experimental tests, we found that the experimental results are optimal when the coefficients $\lambda_{1}$ and $\lambda_{2}$ are set to 100.

## 3. Experimental Results and Discussion

### 3.1. Measurement System Setup

To validate the effectiveness of our method, we constructed the FPP system comprising a 1920 × 1080 pixels projector (SHENGFENG TECH, Shenzhen, China) equipped with a 35 mm lens and a 3072 × 2048 pixels camera (HIKVISION, Hangzhou, China), fitted with a low-aberration telecentric lens (model MVL-MY-045-135C-MP) (HIKVISION, Hangzhou, China). To obtain encoded fringe images devoid of scattering effects for the translucent objects as ground truth images, the surfaces of these objects were powder-coated using AESUB 3D scanning powder (STATE OF THE ART SCANNING SPRAY, Recklinghausen, Germany). This coating minimized the impact of scattering on the fringe patterns. It is worth noting that the spray used in this study evaporated automatically after a few hours. Both coated and uncoated objects were measured in the same position and without any movement, ensuring that the coordinates remained consistent between the two measurements.

**Figure 6:**
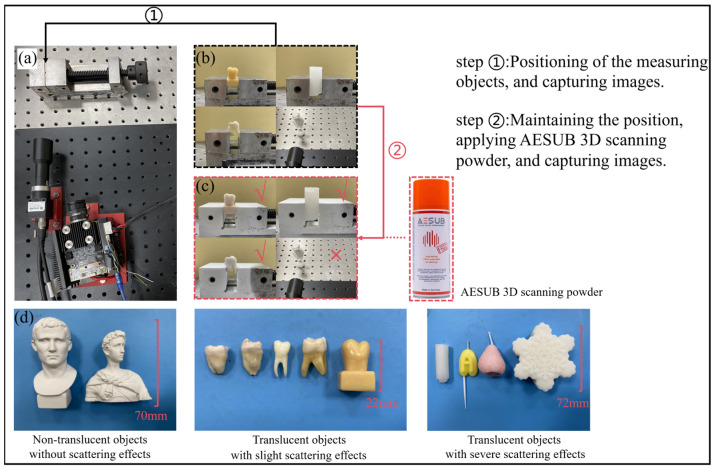
Schematic diagram of the experimental data acquisition process. (**a**) Setup of the fixture and camera for object fixation. (**b**) The four measurement objects used in the experiment. (**c**) Application of AESUB 3D scanning powder and image acquisition. (**d**) Three types of partial measurement objects.

The schematic diagram of the experimental data acquisition process is shown in Figure 6. In Figure 6a, a fixture is placed in front of the camera to secure the measurement object. During data acquisition, the first step involves fixing the object to the fixture, followed by projecting a set of patterns and synchronously triggering the camera to capture images. Figure 6b illustrates the four measurement objects used in the experiment. In the second step, the object’s position is maintained, and AESUB 3D scanning powder is evenly sprayed at a distance of 15–20 mm from the object to capture the second set of images, as shown in Figure 6c. Since the plaster object is a uniformly reflective surface, no spraying is required. Figure 6d displays three types of partial measurement objects used in the experiment. Due to the limitations of the hardware system, the camera’s field of view is 30 mm × 30 mm, meaning only local regions of larger objects can be captured.

### 3.2. Training Implementation Details

A total of 1200 datasets were collected for various scenes, including translucent objects with both slight and severe scattering effects, as well as plaster objects. The dataset was divided into training, validation, and test sets. Notably, the training set included all datasets with a fringe frequency of 64, while the test set comprised datasets with fringe frequencies of 32 and 16. The selection of frequencies was primarily determined by the Gray code-based phase unwrapping algorithm. During the experiments, the unwrapped phase obtained by combining the 12-step phase-shifting method with complementary Gray codes was used as the ground truth. The number of bits in the Gray code must correspond to the number of fringe periods. To ensure reliable measurement accuracy within the system’s operating parameter range, frequencies of 16, 32, and 64 were selected for the experiments.

To clearly demonstrate the generalization capabilities and advantages of our proposed networks, we incorporated an additional publicly available dataset [10] for training and conducted a comprehensive comparisons of the results. For clarity and subsequent reference, the publicly available dataset is named Dataset 1, while our self-built dataset is named Dataset 2. For Dataset 1, which consists of plaster objects, the number of training datasets is 800, the number of validation sets is 50, and the number of test sets, named Test A, is 150. Regarding Dataset 2, the training set contains 1000 datasets, the validation set is composed of 97 datasets, and the test set is further divided into three categories. Test 1 pertained to 29 objects that exhibited no scattering effects, more specifically, gypsum objects. Test 2 involved 50 translucent objects with slight scattering effects, and Test 3 captured 24 translucent objects with severe scattering effects. Detailed information regarding the two datasets is provided in Table 1.

**Figure 7:**
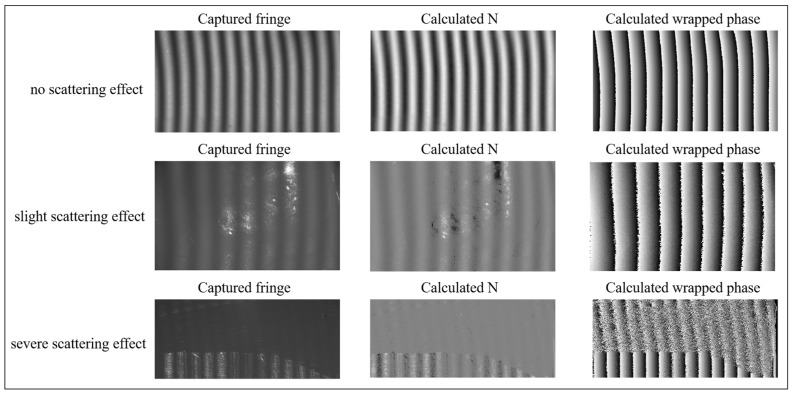
Fringe images with different degrees of scattering effects.

In this study, to distinguish between slight and severe scattering effects, we employed the Structural Similarity Index Measure (SSIM) to quantitatively assess the degree of scattering effects. SSIM is a widely employed metric in image quality assessment, effectively measuring the structural similarity between two images. Due to the varying degrees of scattering effects, the wrapped phase map exhibits different levels of degradation. This characteristic enables us to compute the differences between wrapped phase maps affected by scattering and a reference wrapped phase map, thereby quantifying the scattering degree of the object. More specifically, images of the tested object were captured before and after powder spraying—the former exhibiting scattering effects and the latter being free from scattering effects. Wrapped phase calculations were then performed on these images, followed by SSIM computations on the resulting wrapped phases. Based on the results from all tested objects, we established the following criteria: SSIM values within [0,0.6] indicate severe scattering, SSIM values within [0.6,0.88] suggest slight scattering, and SSIM values within [0.9,1] denote negligible scattering effects. Fringe images with different degrees of scattering effects are shown in Figure 7. It can be observed that when a slight scattering effect exists on the object’s surface, the image acquired by the camera becomes blurred, and the degraded fringe pattern introduces significant phase errors into the wrapped phase. Especially in cases of severe scattering, the fringe image experiences severe attenuation, and the wrapped phase is significantly distorted, leading to inaccurate 3D shape reconstruction. Therefore, the objective of this paper is for the proposed network to obtain correct wrapped phase information, even when using degraded fringe images.

All networks were trained on NVIDIA RTX 4090 GPU (NVIDIA, Santa Clara, CA, USA). The initial learning rates for the Adam optimizer were set separately for the generator and the discriminator, with values of $2\times 10^{-4}$ and $5\times 10^{-4}$, respectively.

While the object depth can be easily mapped from the phase information in FPP, we use phase accuracy to evaluate the algorithm’s reconstruction precision. More specifically, in the conducted ablation and comparative experiments, the unwrapped phases derived from the 12-step phase-shifting and complementary Gray code were utilized as the ground truth values. For translucent objects, the results obtained from images treated with AESUB 3D scanning powder were employed as the reference values [28]. In this experimental configuration, two evaluation metrics were adopted as follows: the mean absolute error (MAE) and root mean square error (RMSE), with smaller MAE and RMSE values being preferred. This approach ensures a robust and comprehensive assessment of the predicted results, with a similar program of assessment indicators having been reported in References [29,30,31].

### 3.3. Ablation Studies

#### 3.3.1. Network Framework and Backbone Network

**Figure 8:**
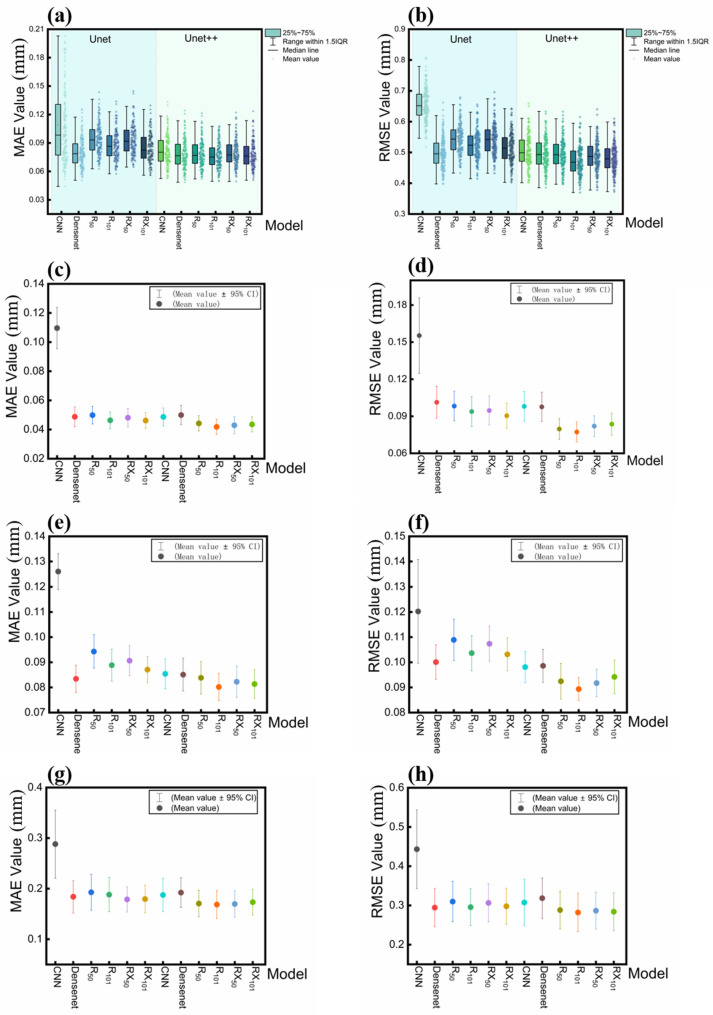
Comparative results of the absolute phases obtained on two datasets with different frames and backbone networks. (**a**,**b**) Boxplots of the 12 models on the Dataset 1 test set. (**c**,**d**) Mean plots of the 12 models on Test 1 of Dataset 2. (**e**,**f**) Mean plots of the 12 models on Test 2 of Dataset 2. (**g**,**h**) Mean plots of the 12 models on Test 3 of Dataset 2.

To validate the efficacy of the generator employing the U-net++ architecture with the backbone network replaced by the ResNet101 structure, we conducted a comparative analysis by combining U-Net++ and U-Net with six classical and mainstream backbone networks (CNN (original network) [22], DenseNet [32], ResNet-50, ResNet-101 [33], ResNeXt-50, and ResNeXt-101 [34]), based on Dataset 1 and Dataset 2. The quantitative outcomes of these two network frameworks and their corresponding backbone replacements are presented in Table 2. Additionally, Figure 8 illustrates the MAE and RMSE results for the two datasets across different models. Figure 8a,b showcases the box plots derived from 150 samples of Dataset 1, effectively highlighting the data’s core distribution characteristics, central tendency, skewness, and the presence of outliers. Figure 8c,d presents the interval plots of the MAE and RMSE values for the 29 non-scattering samples from Test 1 in Dataset 2, emphasizing the mean error values and error ranges. The same interval plots for Test 2 (50 samples with slight scattering) and Test 3 (29 samples with severe scattering) in Dataset 2 are shown in Figure 8e,f and Figure 8g,h, respectively.

Compared with the U-net structure, the MAE and RMSE of the six backbone networks associated with the U-net++ structure are significantly lower, as shown in Table 2. This indicates that the U-net++ structure achieves superior performance compared to the U-net structure. As for the U-net framework, the performance of the CNN backbone obtains the highest MAE and RMSE values, and DenseNet achieves the lowest MAE and RMSE values, as shown in Figure 8a,b. The lowest errors for DenseNet can be attributed to the dense connectivity of its architecture, which allows each convolution layer to be directly connected to all other layers, facilitating better feature information fusion. For both ResNet and ResNeXt, the 101-layer architectures demonstrate superior performance compared to their 50-layer counterparts. As for the U-net++ framework, ResNet101 serves as the most effective backbone network in Figure 8c–h. This is likely due to the multi-layer feature fusion characteristics of the U-net++ structure, which enable rich features to be extracted from the deep architecture of ResNet101 to be integrated effectively across layers, thereby diminishing the relative advantage of DenseNet’s dense connectivity. ResNeXt101 with grouped convolution does not obtain better results than ResNet101. Therefore, the combination of the U-net++ architecture and ResNet101 is identified as the optimal choice. In all subsequent ablation experiments, the U-net++ architecture and ResNet101 feature extraction network were employed.

#### 3.3.2. Effectiveness of Multilevel Attention Fusion Module

**Figure 9:**
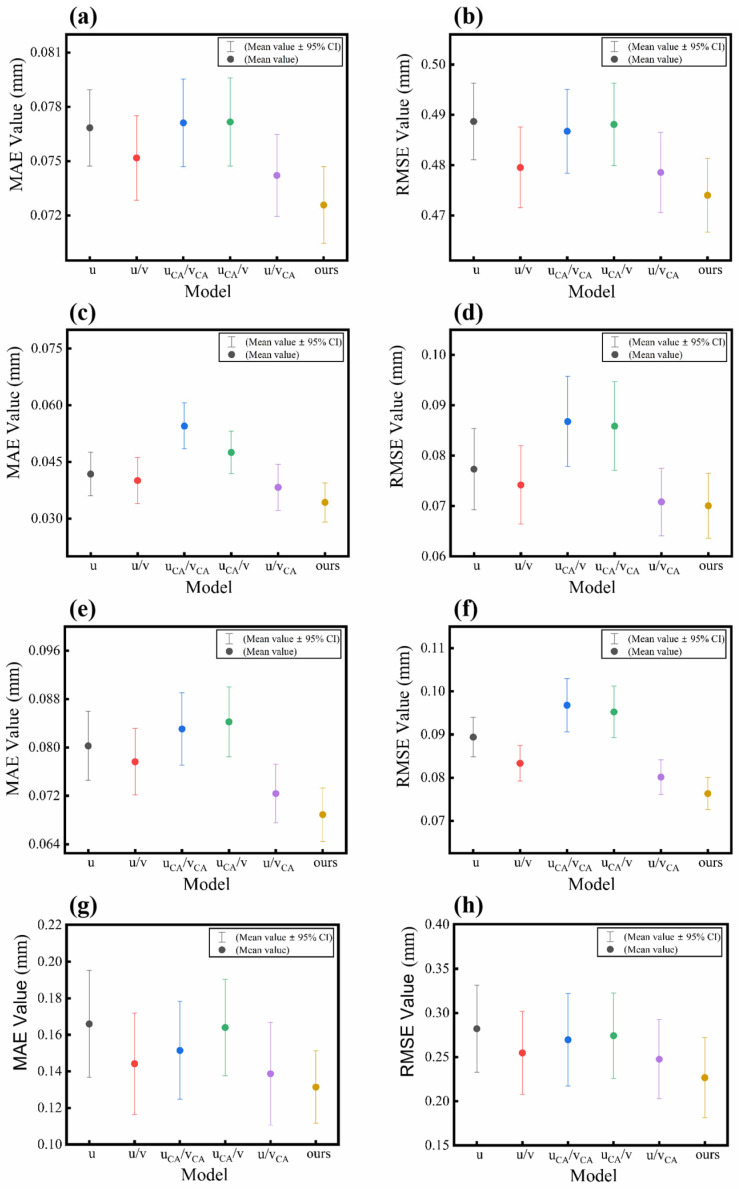
Mean plots of MAE and RMSE based on two datasets for six models. (**a**,**b**) Mean plots of the six models evaluated on the test set of Dataset 1. (**c**,**d**) Mean plots of the six models for Test 1 of Dataset 2. (**e**,**f**) Mean plots of the six models for Test 2 of Dataset 2. (**g**,**h**) Mean plots of the six models for Test 3 of Dataset 2. The description of each model is provided in Table 3.

The effectiveness of the Multilevel Attention Fusion Module (MAFM) is verified through the analysis of the following two distinct aspects: with/without the pre-trained VGG19 network and the attention mechanism’s existence and location. This validation encompassed five distinct models and their mean MAE and RMSE plots, which Figure 9 depicts. At the same time, Table 3 provides a detailed description of each model and its corresponding acronym.

1. Pre-trained VGG-19 Network

The pre-trained VGG-19 network, serving as a crucial component of the MAFM, assumes a pivotal role in enhancing the overall reconstruction quality. To comprehensively illustrate the effectiveness of the pre-trained VGG-19 network, we conducted a comparative analysis utilizing objective evaluation metrics on two datasets, as presented in Table 4. Upon reviewing the data in Table 4, it becomes evident that the absence of the pre-trained VGG19 network results in an increase in MAE and RMSE for Dataset 1 and Dataset 2, which can also be observed in Figure 9a–h, as follows: the mean values of the u model are consistently higher compared to those of the u/v model. In the u/v model, the MAE/RMSE values for Dataset 1 in Test A and Dataset 2 in Test 1, Test 2, and Test 3 are reduced by 2.16%/1.87%, 4.20%/4.07%, 3.26%/6.75%, and 9.67%/13.11%, respectively, compared to those in the u model. Notably, the errors of Test 3 demonstrated the most significant reduction subsequent to the integration of the pre-trained VGG19 network. This observation underscores the enhanced efficacy of the pre-trained VGG19 network in mitigating the issue of information loss attributable to severe scattering, thereby improving the accuracy of network predictions for Test 3.

2. Attention Mechanism

Table 5 presents the results for the two datasets with different CA locations. As indicated in Table 5, when the CA is only added to the pre-trained VGG-19 branch, the MAE and RMSE values decrease, which can also be observed in Figure 9a–h, as follows: the mean values of the $u/v_{CA}$ model are consistently lower compared to those of the u/v model. This observation indicates that adding CA to the pre-trained VGG-19 branch can enhance the accuracy of the unwrapped phase.

However, based on the analysis presented in Table 5, it is evident that the MAE and RMSE values for both the $u_{CA}/v$ and $u_{CA}/v_{CA}$ models are higher than those for the u/v model, which can also be visualized in Figure 9a–g. This suggests that the addition of a CA to the backbone branch results in elevated error values, thereby diminishing the performance of the network. This means that CA should not be added across all branches of MAFM simultaneously, but only to the pre-trained VGG-19 branch. Within the network, there are three MAFM located in three different layers. Through further experiments, the impact of CA’s presence at different levels on the network prediction results was investigated and is provided as supplementary information. As shown by the data in Table S1, the network achieves optimal performance when the CA mechanism is added to all three layers of the network. Consequently, in the proposed network architecture, CA is incorporated into all MAFMs and specifically applied to the pre-trained VGG-19 branch.

#### 3.3.3. Impact of Loss Function Design

To verify the effectiveness of the generator loss function with the incorporation of Mean Squared Error (MSE) loss, we conducted a comparison between networks with/without the MSE loss function. This comparison is based on the optimal configurations utilizing the pre-trained VGG19 network and the attention mechanism (CA), as indicated by the results of the aforementioned ablation experiments. As shown in Table 6, the comparison results were obtained on two datasets with/without MSE loss for the absolute phases. More specifically, with MSE loss, the MAE values for Dataset 1 in Test A and Dataset 2 in Test 1, Test 2, and Test 3 are reduced by 1.20%, 10.70%, 5.8%, and 2.90%, compared to those without MSE loss. As we can see in Figure 9a–h, our model has the lowest mean values among the six models. These results demonstrate that the incorporation of MSE loss positively influences network performance.

### 3.4. Comparison Experiments

To highlight the advantages of the proposed algorithms, we conducted a comparative analysis between the following three methods: Unet-Phase [10], CDLP [35], and DCFPP [29], all of which utilize a single frame of images for predicting wrapped phase information. The accuracy of the unwrapped phases computed by these three algorithms was evaluated to assess their performance. In our comparative experiments, the peak signal-to-noise ratio (PSNR) was also adopted as an evaluative criterion. As one of the reliable quantitative metrics for measuring the discrepancy between the original data and the predicted data, PSNR provides a robust standard for assessing the fidelity of phase reconstruction (with a larger value of PSNR being preferred).

Table 7 and Table 8 present the statistical measurements of four different methods using two datasets, highlighting the best evaluation values. The results clearly demonstrate that the proposed method consistently outperforms the other methods, achieving the highest performance on both Dataset 1 and Dataset 2. From the evaluation metrics, it is evident that the performance of the other three methods follows the order of DCFPP > Unet-Phase > CDLP. Compared to DCFPP, our method shows significant improvements, as follows: the MAE/RMSE values decreased by 28.28%/12.21%, 50.50%/57.21%, 13.53%/23.38%, and 53.52%/41.47% on Test A, Test 1, Test 2, and Test 3, respectively. Additionally, the PSNR values increased by 8.43%, 67.51%, 18.54%, and 35.81% on the same tests.

For objects without scattering effects (e.g., Test A and Test 1), the relatively low PSNR values are primarily caused by the high RMSE values, which stem from the dataset characteristics or the morphological properties of the collected objects. Since PSNR is calculated based on MSE, and RMSE is the square root of MSE, the elevated RMSE directly results in reduced PSNR.

**Figure 10:**
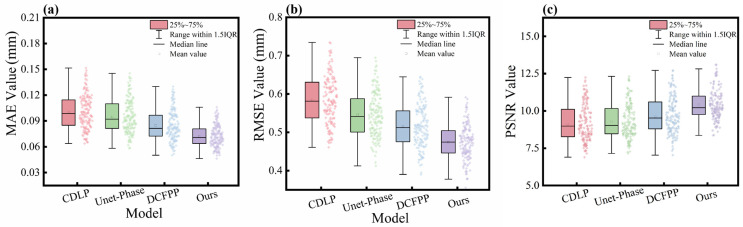
Boxplots corresponding to the MAE (**a**), RMSE (**b**), and PSNR (**c**) for the four methods on Dataset 1.

Figure 10 presents the statistical results of MAE, RMSE, and PSNR for different models on Test A Dataset 1. The range of the box spans from the 25th to 75th percentile. In Figure 10a, the box ranges for CDLP, Unet-Phase, DCFPP, and our method are 0.084–0.115, 0.082–0.11, 0.076–0.095, and 0.065–0.082, respectively. Notably, our method achieves lower values with a narrower range, along with a lower median MAE, indicating higher accuracy and stability. In Figure 10b, which displays the RMSE statistics, our method exhibits the lowest mean and median values, with the shortest box range of approximately 0.45–0.50. In contrast, the box ranges for the other three methods are 0.54–0.63, 0.50–0.59, and 0.47–0.55, respectively, exhibiting broader distributions and longer boxes. Our method’s relatively concentrated distribution underscores its superior stability. In Figure 10c, where higher PSNR values signify better predictive performance, the box ranges for the four methods (from left to right) are 8.20–10.05, 8.45–10.10, 8.90–10.85, and 9.9–11, respectively. Evidently, our method’s box plot is positioned at the highest level, showing a more concentrated data distribution. It outperforms the other methods in both median and mean PSNR values, with fewer outliers, highlighting its robust performance. For CDLP and Unet-Phase, the overall distribution is positively skewed, with a small portion of the data exceeding a PSNR of 10, but with the majority of the data clustered around 9.

Overall, our method outperforms CDLP, Unet-Phase, and DCFPP in all metrics within the publicly available datasets, demonstrating superior accuracy, consistency, and stability.

**Figure 11:**
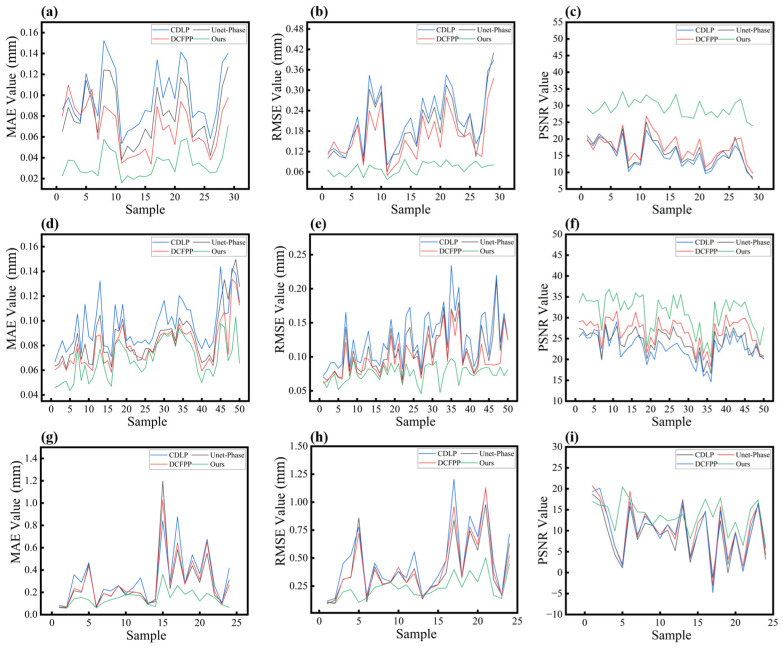
Result curves of MAE, RMSE, and PSNR on Dataset 2 for the four methods. (**a**–**c**) Curves of MAE, RMSE, and PSNR on Test 1 (objects without scattering effects). (**d**–**f**) Curves of MAE, RMSE, and PSNR on Test 2 (objects with slight scattering effects). (**g**–**i**) Curves of MAE, RMSE, and PSNR on Test 3 (objects with severe scattering effects).

Figure 11 shows the curves based on Dataset 2 of Test 1 (29 non-translucent samples without scattering effect), Test 2 (50 translucent samples with slight scattering), and Test 3 (24 translucent samples with severe scattering), respectively. These curves show that our method consistently achieves the best results in the datasets collected by our team, exhibiting smoother performance compared to other methods.

(1) Non-translucent objects without scattering effects:

Our method’s superiority on non-transparent objects has been validated on public datasets, and the curves in Figure 11a–c further corroborate this conclusion. More specifically, the green curves representing MAE and RMSE are positioned at the lowest levels, with the RMSE curve exhibiting a relatively smooth trend. Additionally, the PSNR values remain consistently high across the samples. These observations demonstrate that the proposed method exhibits high stability and reliability when processing non-transparent objects.

(2) Translucent objects with slight and severe scattering effects:

The application of our proposed method to translucent samples represents a core contribution of this work, as these samples present unique challenges due to scattering effects. Combining the data analysis from Table 8 and Figure 11d–i, for all of the three methods (CDLP, Unet-Phase, and DCFPP), the input images exhibit low modulation, low SNR, and high noise due to varying degrees of scattering effects. This significantly impacts the performance of these networks, leading to a severe reduction in the prediction accuracy of wrapped phase information. As shown in Table 8 (Test 2), for translucent objects with mild scattering, our method achieves average MAE, RMSE, and PSNR values of 0.0690 mm/0.0757 mm/31.7041, respectively, significantly outperforming other comparative methods. For instance, CDLP yields average MAE, RMSE, and PSNR values of 0.0961 mm/0.1269 mm/22.8727, respectively. The errors associated with these methods begin to increase, showing a more dispersed distribution. As illustrated in Figure 11d,e, the MAE and RMSE curves for CDLP remain consistently at the highest levels, followed by those of Unet-Phase and DCFPP.

From Figure 11g–i, when the scattering effects of translucent objects become severe, the error curves of the other three methods exhibit significantly more pronounced fluctuations. As evidenced by the data from Table 8 (Test 3), our method achieves average MAE/RMSE/PSNR values of 0.1314 mm/0.2366 mm/13.5463, respectively, across all test samples. In contrast, CDLP, Unet-Phase, and DCFPP yield corresponding values of 0.3192 mm/0.4693 mm/8.9490, 0.2766 mm/0.3927 mm/10.3986, and 0.2827 mm/0.4043 mm/9.9743, respectively. These results clearly demonstrate that the comparative methods exhibit significantly larger MAE and RMSE values. This is primarily attributed to the scattering effects, which disrupt the original data characteristics, and these methods lack adequate mechanisms to handle such complex interference. Some prominent error peaks indicate serious mispredictions, particularly in regions where scattering dominates.

In comparison, our proposed method achieves significantly lower evaluation metric errors and better captures phase information, especially for translucent samples. Notably, for samples significantly affected by scattering, our method maintains relatively low error values. Even under severe scattering conditions, the error distribution of our method remains relatively concentrated. This robustness can be attributed to the specialized modules integrated into our approach, such as the MAFM, which enables more robust feature extraction, even in the presence of scattering interference. As a result, it enables the effective recovery of the damaged phase information. It is worth noting that the training datasets used for all four methods do not include objects with severe scattering effects. Therefore, when testing new objects exhibiting significant scattering effects, our network demonstrates superior generalization and stability compared to the other three methods. This highlights the practical applicability of our method in real-world scenarios where scattering effects are prevalent.

Overall, our method demonstrates superior performance across the majority of samples, particularly for translucent objects. However, for specific individual samples, the results from all four methods are pretty similar. We analyze these cases as follows: when the sample error values for all four methods are low, this likely indicates that the samples were distinct and easily recognizable for all networks; when the error values are high across the board, it suggests that these samples contained significant noise or confusing features, making accurate predictions challenging for all models.

### 3.5. Scene Presentation

Based on the findings of the comparative experiments detailed in Section 3.4, individual samples were extracted from both the public dataset and the self-built dataset to demonstrate the advantages of the proposed method further. These samples were used to compare the performance of CDLP, Unet-Phase, DCFPP, and our proposed method across specific scenarios. The comparison included phase error maps and the corresponding 3D reconstruction results for these scenarios.

#### 3.5.1. Scene Presentation for Public Dataset (Dataset 1)

**Figure 12:**
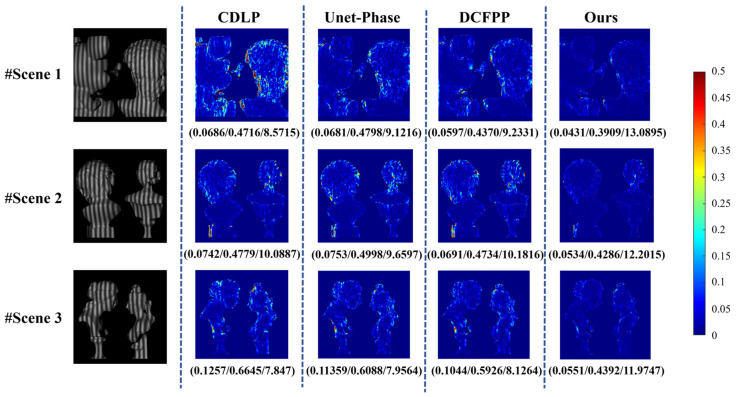
Calculated unwrapped phase maps and errors on Dataset 1. The evaluation index (MAE (mm)/RMSE (mm)/PSNR) for each error map is listed below the image.

In Dataset 1 of Test A, three complex and isolated scenes are selected, and the modulation patterns of these three scenes are depicted in the first column of Figure 12. After obtaining the deformed fringe image, it can be input into the network to obtain the wrapped phase map, and then, the corresponding unwrapped phase map can be obtained through the fringe order.

Figure 12 sequentially displays the unwrapped phase error maps calculated by CDLP, Unet-Phase, DCFPP, and the proposed method, with the MAE/RMSE/PSNR values for each image labeled below the images. Based on these evaluation metrics, it can be observed that the proposed method achieves the lowest MAE and RMSE compared to the other three methods. From the error maps, it is evident that CDLP, Unet-Phase, and DCFPP exhibit significant phase errors at the edges of specific objects. This is likely due to the potential limitations of their convolutional neural network architectures in capturing complex phase variations at the edges. The fixed receptive fields of convolutional layers may not adequately adapt to rapid phase changes.

In contrast, our method consistently maintains lower overall errors and higher accuracy, particularly in edge regions. This is attributed to the multilevel attention fusion module and the U-net++ framework employed in our approach, which enables the capture of both local and global phase information, thereby better handling abrupt phase changes at object edges. This demonstrates that our method can more effectively address phase errors, maintaining high precision even in challenging regions. Owing to the deficiency of crucial data within public datasets, acquiring the corresponding 3D reconstruction outcomes is infeasible.

#### 3.5.2. Scene Presentation for No Scattering Effect in the Self-Built Dataset (Dataset 2)

**Figure 13:**
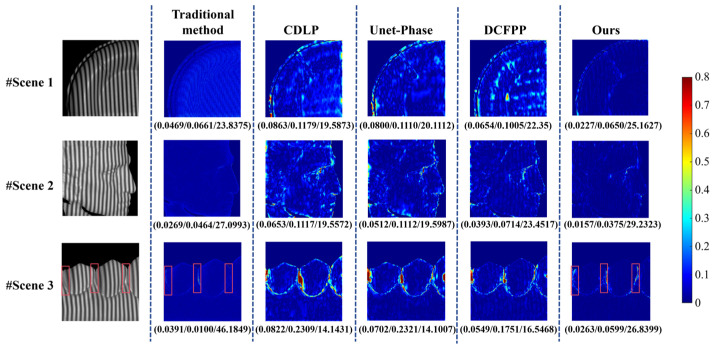
Calculated unwrapped phase maps and errors on Test 1 of Dataset 2. The evaluation index (MAE (mm)/RMSE (mm)/PSNR) for each error map is listed below the image. Red boxes indicate areas with crevices or insufficient fringe pattern illumination.

In Dataset 2 of Test 1, the error maps for the three samples are presented in Figure 13. The layout of these maps is generally consistent with that of Figure 12, with the exception of an additional column presenting the phase error maps computed by the traditional FPP algorithm (four-step phase-shifting method). Overall, the images in the last column exhibit a predominant deep blue coloration, indicating that our method significantly outperforms the other three deep learning methods in reducing errors. This advantage is particularly evident in Scene 3, where our method demonstrates notably more minor and more precise boundary errors on the measured objects compared to the other approaches.

**Figure 14:**
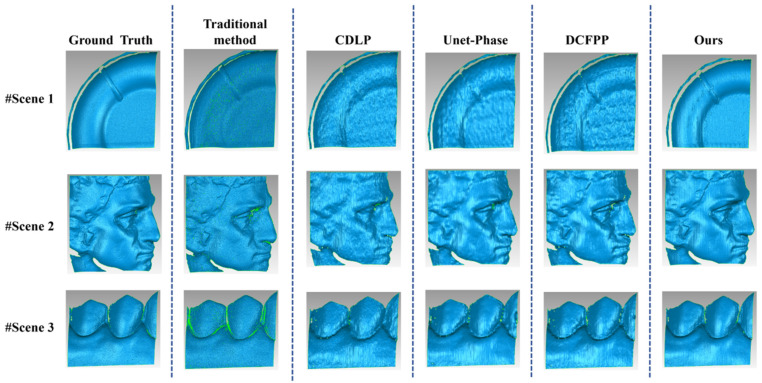
Three-dimensional reconstruction maps of three scenes on Test 1 of Dataset 2.

Figure 14 illustrates the reconstruction results for the three scenes corresponding to each method discussed in Figure 13, obtained using Geomagic Wrap software 2021. The ground truth reconstruction results for the three scenes are shown in the first column of Figure 14, while the second through sixth columns show the reconstruction results for the traditional method, CDLP, Unet-Phase, DCFPP, and our proposed method, respectively. Compared to other deep learning methods, the reconstructions from our method display greater detail and are closer to the ground truth. Although the reconstruction results from the other three methods appear essentially complete overall, they exhibit varying degrees of detail loss. Scenes 1 and 3 display noticeable wrinkling, while the character texture details in Scene 2 have been smoothed and blurred. Among the methods evaluated, the reconstruction results from CDLP are the least satisfactory.

While our deep learning-based approach generally outperforms other deep learning methods, it occasionally exhibits slightly inferior performance compared to the traditional method, particularly in complex local regions such as crevices or areas with insufficient fringe pattern illumination (e.g., Scene 3). This discrepancy can be attributed to the fundamental difference in measurement mechanisms between the two approaches. More specifically, the traditional method relies on four phase-shifted images, which provide richer information for reconstructing complex regions. In contrast, our method operates on a single image, which may struggle to capture fine details in challenging areas, as highlighted in the red box of Scene 3. The implementation of a multi-frame acquisition strategy or the adoption of adaptive illumination techniques could effectively address this limitation. However, it is important to note that our method demonstrates superior performance in regions with relatively smooth surface topography, contributing to an overall lower MAE across the entire measurement. Additionally, as shown in Figure 14, the traditional method still exhibits noticeable fringe-induced ripples in Scene 3, whereas our proposed method produces a 3D reconstruction that more closely approximates the reference values.

#### 3.5.3. Scene Presentation for Slight Scattering Effect in the Self-Built Dataset (Dataset 2)

**Figure 15:**
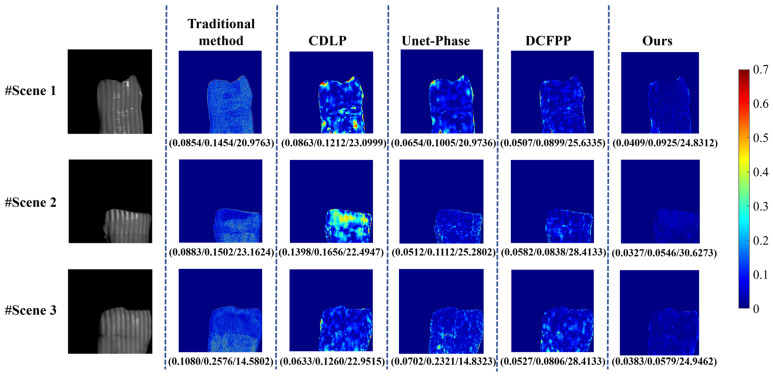
Calculated unwrapped phase maps and errors on Test 2 of Dataset 2. The evaluation index (MAE (mm)/RMSE (mm)/PSNR) for each error map is listed below the image.

Figure 15 presents the measurement results of three translucent objects exhibiting mild scattering effects. In the first column, the fringe patterns on the surfaces of all three measured objects are observed to be blurred due to subsurface scattering effects. The second column displays the error distribution maps obtained using conventional methods, with corresponding MAE/RMSE/PSNR metrics of 0.0854/0.1454/20.9763, 0.0883/0.1502/23.1624, and 0.1080/0.2576/14.5802, respectively. Quantitative analysis reveals that the conventional approach generates substantial and dense phase errors across all three scenarios, demonstrating the impact of scattering effects on phase measurement accuracy in translucent objects. This phenomenon can be attributed to the oversimplified light–object interaction model employed by conventional methods, which exhibits high sensitivity to surface scattering effects, leading to light path deviation and consequent phase distortion.

In contrast, the proposed method demonstrates significant improvements, achieving MAE/RMSE/PSNR values of 0.0409/0.0925/24.8312, 0.0327/0.0546/30.6273, and 0.0383/0.0579/24.9462, respectively. The error maps show minimal deviations, predominantly represented by deep blue regions approaching zero. These results confirm the effectiveness of the proposed method in recovering measurement phases of translucent objects while maintaining low error levels. The performance enhancement is primarily attributed to the improved model developed in this study, which incorporates scattering-induced phase error correction through training. More specifically, the model achieves precise scattering estimation by analyzing local fringe characteristics, enabling the accurate reconstruction of the wrapped phase and a substantial reduction in overall measurement errors. It should be noted that while the other three comparative deep learning methods demonstrate particular effectiveness in reducing overall measurement errors, their error distribution maps reveal noticeable local defects. The CDLP method mainly exhibits significant error concentration areas in Scene 1 and Scene 2. Quantitative analysis indicates that in these scenes, the CDLP method yields MAE values of 0.0863 and 0.1398, respectively, compared to 0.0409 and 0.0327 achieved by the proposed method. This comparative result suggests the limited performance of the CDLP method in handling mild scattering scenarios, potentially due to its network architecture not being explicitly optimized for scattering-induced error patterns.

**Figure 16:**
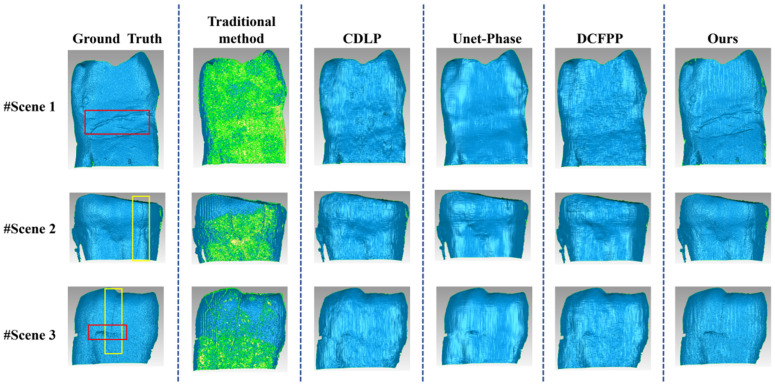
Three-dimensional reconstruction maps of three scenes on Test 2 of Dataset 2. The red boxes indicate the textured areas, and the yellow boxes indicate the crack areas.

Figure 16 presents the three-dimensional reconstruction results corresponding to Figure 15, maintaining the same layout as Figure 14. The reconstruction results obtained using conventional methods, shown in the second column, reveal extensive green regions on the surfaces of the three translucent objects. These regions reflect phase errors and noise interference induced by mild scattering effects, resulting in inaccurate point cloud reconstruction. In contrast, all four deep learning methods demonstrate improved surface encapsulation, though significant variations in reconstruction quality are observed. More specifically, the surfaces reconstructed by the CDLP and DCFPP methods exhibit noticeable roughness, particularly in Scene 1, where irregular undulations are present. Although the Unet-Phase method achieves relatively smooth surface reconstruction, excessive smoothing leads to the loss of original detail features. Comparatively, the proposed method, despite showing slight surface ripples, demonstrates superior fidelity in reproducing overall detail textures, closely approximating the reference values. This advantage is particularly evident in the texture regions marked by red boxes and crack areas indicated by the yellow boxes in Figure 16, where the proposed method successfully preserves these critical detail features. In contrast, the CDLP, Unet-Phase, and DCFPP methods fail to reconstruct them fully. The experimental results indicate that while conventional methods are unable to reconstruct translucent objects with mild scattering effects accurately, the proposed method achieves satisfactory reconstruction outcomes. Although the other three deep learning methods accomplish essential surface reconstruction, they exhibit notable deficiencies in preserving detailed features. This finding further confirms the superiority and robustness of the proposed method in scenarios involving translucent objects with scattering effects.

#### 3.5.4. Scene Presentation for Severe Scattering Effect in the Self-Built Dataset (Dataset 2)

**Figure 17:**
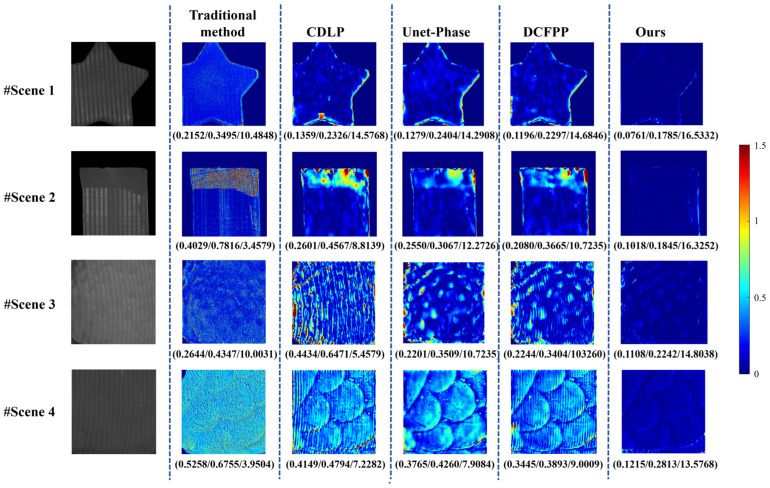
Calculated unwrapped phase maps and errors on Test 3 of Dataset 2. The evaluation index (MAE (mm)/RMSE (mm)/PSNR) for each error map is listed below the image.

The error evaluation results for four translucent objects exhibiting severe scattering effects can be seen in Figure 17, where Scene 1 and Scene 2 possess relatively simple geometric configurations, while Scene 3 and Scene 4 demonstrate more complex structural characteristics. The fringe patterns in the first column clearly show significant blurring and drastic contrast reduction due to severe scattering effects. Compared with the results from Test 1 and Test 2, all five evaluation methods exhibit varying degrees of increased error metrics. The traditional method demonstrates poor performance under severe scattering conditions, with MAE/RMSE/PSNR values of 0.2152/0.3495/10.4848, 0.4029/0.7816/3.4579, 0.2644/0.4347/10.0031, and 0.5258/0.6755/3.9504 for the four scenes, respectively. The error distribution maps, with a color bar range of [0,1.5], reveal extensive high-error regions in Scene 2 and Scene 4. These results clearly indicate the substantial limitations of conventional methods in handling severe scattering scenarios, failing to maintain measurement accuracy and reliability under complex conditions. The comparative methods also show unsatisfactory performance. In Scene 1, all three comparative methods exhibit significant errors at object edges; Scene 2 displays high-error characteristics in severely blurred regions; and Scene 4 shows only minimal areas with the ideal dark blue in the reconstruction results. Among these, the CDLP method faces the most significant challenges, with MAE/RMSE values of 0.1359/0.2326, 0.2601/0.4567, 0.4434/0.6471, and 0.4149/0.4794 for the four scenes, respectively, indicating its difficulty in accurately reconstructing complex-shaped objects.

In contrast, the proposed method demonstrates significant advantages. As shown in the last column of Figure 17, the phase error maps present relatively uniform dark blue regions without noticeable high-error areas. This result confirms that even under severe surface scattering conditions, the proposed method maintains strong phase information prediction capability and low error metrics. The experimental data visually demonstrate the accuracy and reliability of the proposed method in translucent object measurement, highlighting its exceptional performance in handling challenging scattering scenarios. Compared with the traditional method, the effectiveness of the proposed method under complex scattering conditions has been thoroughly validated.

**Figure 18:**
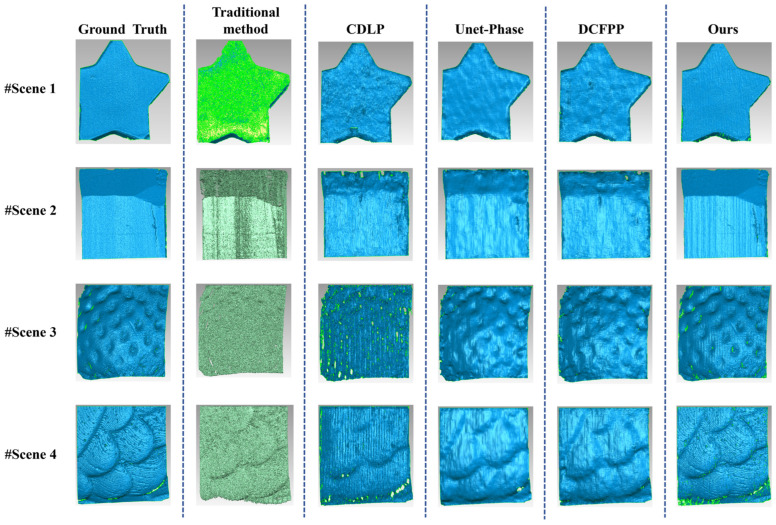
Three-dimensional reconstruction maps of four scenes based on Test 3 of Dataset 2.

The 3D reconstruction results of the four translucent objects described in Figure 17 are presented in Figure 18. The first column displays the reference results for each scene, which were obtained by employing 3D scanning powder to mitigate scattering effects. The second column illustrates the reconstruction results using the conventional FPP algorithm. In Scene 1, correct encapsulation could not be achieved due to severe noise interference, while in Scenes 2, 3, and 4, incomplete point cloud data resulting from strong scattering effects prevented encapsulation. These results clearly reveal the inherent limitations of the conventional FPP algorithm in handling translucent objects with significant scattering effects. Although the CDLP, Unet-Phase, and DCFPP methods are capable of obtaining complete wrapped phase information and achieving essential three-dimensional reconstruction, their reconstruction quality remains notably inadequate. More specifically, in Scene 2, these methods fail to reconstruct the geometric shapes of the severe scatter effect regions accurately; in Scene 3 and Scene 4, while the Unet-Phase and DCFPP methods partially restore the basic features of the objects, they still lose substantial surface details, and the reconstructed surfaces exhibit excessive smoothing. Among these, the CDLP method demonstrates the poorest reconstruction performance, failing to preserve the key features of the objects effectively.

In contrast, the proposed method achieves significantly superior reconstruction results. Experimental results indicate that this method effectively preserves the surface texture features of translucent objects. This performance advantage is primarily attributed to the following factors: the improved model possesses robust information feature extraction capabilities; the network architecture achieves comprehensive information fusion; and the introduction of the MAFM enables the model to effectively process highly blurred fringe information, thereby accurately predicting phase information. These technical enhancements collectively ensure the excellent performance of the proposed method under complex scattering conditions for translucent objects.

### 3.6. Performance Analysis Under the Influence of Different Noises

To evaluate the noise resistance of the proposed method, Gaussian white noise with varying signal-to-noise ratios (SNRs) was introduced to Dataset 1 for experimentation. The MAE/RMSE curves under different noise levels are illustrated in Figure S1. The results indicate that CDLP, Unet-Phase, and DCFPP exhibit significant instability and notably higher errors at lower SNRs. In contrast, the curve representing our method demonstrates a more stable trend compared to the other three, highlighting its superior robustness against interference across different noise levels. Detailed experimental procedures, results, and corresponding figures (Figure S1) are provided as supplementary information.

### 3.7. Performance Analysis Under the Influence of Different Fringe Frequencies

As previously noted, all networks were trained using a 64-frequency fringe. In this section, to assess the generalization performance of the networks, we presented two unseen scenes of the four networks, employing fringe frequencies of 64, 32, and 16. Figure S2 illustrates the reconstruction results of the four deep-learning methods across these frequencies. The results demonstrate that the proposed method outperforms CDLP, Unet-Phase, and DCFPP in terms of reconstruction accuracy and robustness across the three fringe frequencies, exhibiting superior generalization performance. Detailed experimental results, including the reconstructed outcomes and comparisons with other methods, are provided in the supplementary information.

### 3.8. Accuracy Evaluation

In structured light measurement systems, the use of spherical and planar objects for assessing accuracy is a widely adopted practice. To further validate the precision of our algorithm, a standard evaluation was conducted by measuring a standard ceramic plate and a standard ceramic sphere with a radius of 3.25 mm. The relevant data results are shown in Figure S3, where the MAE/RMSE values for the fitted plane and sphere are 0.0212/0.0308 and 0.0527/0.0633, respectively. Compared to the method reported by Li et al. [29], our approach achieves a lower RMSE value, demonstrating that high-quality 3D measurement results can be effectively obtained using only a single fringe image.

## 4. Conclusions

In this study, we present a deep learning 3D measurement technology, which is capable of accurately acquiring wrapped phase information using only a single fringe image, offering a novel approach for the surface measurement of translucent objects. Through comprehensive experimental validation, our proposed method demonstrates exceptional performance in measuring translucent objects affected by varying degrees of scattering phenomena. The technique effectively mitigates disturbances caused by surface scattering, enabling the extraction of more accurate and comprehensive phase information. This achievement is attributed to the proposed GAN-PhaseNet generator, which integrates a U-net++ architecture, with ResNet101 as its backbone, and incorporates a multilevel feature fusion module. Within this module, a pre-trained VGG19 network, enhanced by a channel attention mechanism, significantly improves the extraction of source image feature information. This enhancement not only strengthens the network’s feature extraction capabilities but also augments its spatial feature extraction accuracy, particularly in capturing inter-pixel gradient information. Furthermore, the generator’s loss function incorporates an MSE loss to improve the network’s sensitivity to outliers, thereby enhancing its robustness. The experimental results underscore the unique advantages and specificity of our proposed method in addressing the surface scattering challenges associated with translucent objects. Additionally, the method achieves high precision in measuring gypsum objects with uniform reflective properties, further demonstrating its versatility and effectiveness.

Although our method has demonstrated superior performance in scenarios involving scattering effects, it still possesses certain limitations. Firstly, the model’s training time and computational cost are relatively high. GAN-PhaseNet contains a large number of parameters, resulting in a lengthy training process (approximately 20 h on a single NVIDIA 4090 GPU). Table 9 presents the time consumption for testing 150 objects using each method, with GAN-PhaseNet requiring the longest duration, which limits its application in tasks demanding high real-time performance. Secondly, the data collection process for training is cumbersome. The dataset used in this experiment necessitates the application of AESUB 3D scanning powder to form a thin coating of approximately 7 μm on the object’s surface to eliminate scattering effects. Although this coating has a minimal impact on shape measurement, its application and removal processes significantly increase the complexity of data acquisition. Additionally, the current method still requires improvements when measuring challenging regions. In future work, we plan to extend our approach to handle discontinuous fringe patterns, which are commonly encountered in dynamic or complex surface measurements. To address these limitations, we plan to make advancements in the following aspects:

(1) Model optimization: Reduce the number of model parameters through pruning algorithms and explore quantization techniques (such as dynamic quantization [35] and quantization-aware training [36]) to lower computational complexity, thereby enhancing operational speed and efficiency.
(2) Data augmentation: Employ algorithm-based scattering error correction techniques to minimize dependence on AESUB coatings, streamline the data collection process, and enhance dataset diversity.
(3) Multi-view input: Input multiple single-frame images captured from different angles into the network, leveraging multi-view information to strengthen the understanding of the object’s three-dimensional structure, further improving measurement accuracy and robustness.
(4) Physical model integration: Explore the integration of physical models with deep learning to more effectively handle scattering effects while reducing dependence on large-scale training data.

In summary, while the current method has made significant progress in phase retrieval and 3D reconstruction, further optimization is needed in terms of computational efficiency, data collection, and model accuracy capabilities. Future work will focus on addressing these challenges to promote the widespread application of this method in practical scenarios.

## Figures and Tables

**Table 1:** Collected object information for Dataset 1 and Dataset 2.

Dataset’s Name (Total Number) (Source)	Classification of Datasets (Number)	Collection Objects
Dataset 1 (1000) (publicly available [10])	Train (800)	Gypsum
	Validation (50)	Gypsum
	Test A (150)	Gypsum
Dataset 2 (1200) (self-built)	Train (1000)	Gypsum and objects with a slight scattering effect
	Validation (97)	Gypsum and objects with a slight scattering effect
	Test 1 (29)	Gypsum
	Test 2 (50)	Objects with a slight scattering effect
	Test 3 (24)	Objects with severe scattering effects

**Table 2:** Comparative results of absolute phases obtained on two datasets from different frames and backbone networks.

Method		Dataset 1		Dataset 2					
		Test A		Test 1		Test 2		Test 3	
Frame	Backbone	MAE (mm)	RMSE (mm)	MAE (mm)	RMSE (mm)	MAE (mm)	RMSE (mm)	MAE (mm)	RMSE (mm)
U-net	CNN	0.1139	0.6557	0.1096	0.1553	0.1276	0.1202	0.2835	0.4433
	DenseNet	0.0802	0.4987	0.0487	0.1010	0.0834	0.1000	0.1831	0.2944
	ResNet-50	0.0939	0.5432	0.0498	0.0982	0.0951	0.1089	0.1908	0.3098
	ResNet-101	0.0876	0.5236	0.0463	0.0938	0.0888	0.1036	0.1868	0.2956
	ResNeXt-50	0.0936	0.5414	0.0413	0.0946	0.0906	0.1073	0.1751	0.3066
	ResNeXt-101	0.0850	0.5137	0.0462	0.0904	0.0854	0.1032	0.1764	0.2977
U-net++	CNN	0.0823	0.5052	0.0487	0.0980	0.0871	0.0981	0.1848	0.3074
	DenseNet	0.0785	0.4956	0.0479	0.0976	0.0821	0.0986	0.1826	0.2871
	ResNet-50	0.0786	0.4942	0.0442	0.0797	0.0839	0.0925	0.1681	0.2883
	ResNet-101	0.0768	0.4806	0.0418	0.0773	0.0803	0.0887	0.1659	0.2820
	ResNeXt-50	0.0793	0.4886	0.0429	0.0820	0.0822	0.0917	0.1679	0.2840
	ResNeXt-101	0.0780	0.4816	0.0435	0.0837	0.0813	0.0942	0.1693	0.2866

**Table 3:** Detailed descriptions and acronyms of the five models validated with the MAFM.

Model’s Acronym	Description of Model
u	Network without the pre-trained VGG-19, without CA
u/v	Network with the pre-trained VGG19, without CA
$u_{CA}/v_{CA}$	Two branches in the MAFM include CA
$u_{CA}/v$	Backbone branch with CA, the VGG-19 branch without CA
$u/v_{CA}$	Backbone branch without CA, the VGG-19 branch with CA

**Table 4:** Comparison results obtained on two datasets with/without pre-trained VGG19 for absolute phases.

Model Name	Method	Dataset 1		Dataset 2					
		Test A		Test 1		Test 2		Test 3	
		MAE (mm)	RMSE (mm)	MAE (mm)	RMSE (mm)	MAE (mm)	RMSE (mm)	MAE (mm)	RMSE (mm)
u	Without VGG19	0.0768	0.4806	0.0418	0.0773	0.0803	0.0887	0.1659	0.2820
u/v	With VGG19	0.0751	0.4795	0.0400	0.0742	0.0775	0.0827	0.1642	0.2647

**Table 5:** Comparative results obtained on two datasets with different CA positions for absolute phases.

Model Name	CA Position		Dataset 1		Dataset 2					
			Test A		Test 1		Test 2		Test 3	
	u	v	MAE (mm)	RMSE (mm)	MAE (mm)	RMSE (mm)	MAE (mm)	RMSE (mm)	MAE (mm)	RMSE (mm)
u/v			0.0751	0.4795	0.0400	0.0742	0.0775	0.0827	0.1642	0.2647
u/v_CA_		√	0.0742	0.4785	0.0383	0.0708	0.0724	0.0797	0.1637	0.2576
u_CA_/v	√		0.0772	0.4881	0.0545	0.0868	0.0898	0.0966	0.1889	0.2841
u_CA_/v_CA_	√	√	0.0771	0.4867	0.04751	0.0858	0.0830	0.0950	0.1714	0.2796

**Table 6:** Comparison results obtained on two datasets with/without MSE loss for absolute phases.

Model	Dataset 1		Dataset 2					
	Test A		Test 1		Test 2		Test 3	
	MAE (mm)	RMSE (mm)	MAE (mm)	RMSE (mm)	MAE (mm)	RMSE (mm)	MAE (mm)	RMSE (mm)
Without MSE loss	0.0742	0.4785	0.0383	0.0708	0.0724	0.0797	0.1637	0.2576
With MSE loss	0.0725	0.4672	0.0342	0.0700	0.0690	0.0757	0.1314	0.2366

**Table 7:** Measurement results of the four methods on Dataset 1.

Model	Dataset 1		
	Test A		
	MAE (mm)	RMSE (mm)	PSNR
CDLP	0.1115	0.5859	9.1032
Unet-Phase	0.1105	0.5643	9.3049
DCFPP	0.1011	0.5322	9.6079
Ours	0.0725	0.4672	10.4177

**Table 8:** Measurement results of the four methods on Dataset 2.

Model	Dataset 2								
	Test 1			Test 2			Test 3		
	MAE (mm)	RMSE (mm)	PSNR	MAE (mm)	RMSE (mm)	PSNR	MAE (mm)	RMSE (mm)	PSNR
CDLP	0.0980	0.2101	15.2484	0.0961	0.1269	22.8727	0.3192	0.4693	8.9490
Unet-Phase	0.0811	0.1922	16.1272	0.0864	0.1097	24.2756	0.2766	0.3927	10.3986
DCFPP	0.0691	0.1636	17.4531	0.0798	0.0988	26.7437	0.2827	0.4043	9.9743
Ours	0.0342	0.0700	29.2371	0.0690	0.0757	31.7041	0.1314	0.2366	13.5463

**Table 9:** Time consumption of each method for testing 150 objects.

Method	Time (s)
CDLP	16.74
Unet-Phase	22.88
DCFPP	14.91
Ours	37.81

## Data Availability

The data presented in this study can be made available upon reasonable request from the corresponding author.

## Supplementary Materials

The following supporting information can be downloaded at: https://www.mdpi.com/article/10.3390/s25061823/s1, Supplementary Table: Table S1. Comparison results of the absolute phase of CA at different layers on Dataset 1. Supplementary Figures: Figure S1. Evaluation metrics for datasets with different noise levels; Figure S2. Reconstruction results of the four methods at different frequencies; Figure S3. Precision analysis of standard ceramic plate and a standard ceramic sphere. (a, d) 3D reconstruction results by the proposed GAN-PhaseNet. (b, e) Error distribution. (c, f) RMS error.

## References

[1] Zhang S. (2018). High-speed 3D shape measurement with structured light methods: A review. Opt. Lasers Eng..

[2] Dong Z., Mai Z., Yin S., Wang J., Yuan J., Fei Y. (2020). A weld line detection robot based on structure light for automatic NDT. Int. J. Adv. Manuf. Technol..

[3] Furukawa R., Chen E.L., Sagawa R., Oka S., Kawasaki H. (2024). Calibration-free structured-light-based 3D scanning system in laparoscope for robotic surgery. Healthc. Technol. Lett..

[4] Xue J., Zhang Q., Li C., Lang W., Wang M., Hu Y. (2019). 3D Face Profilometry Based on Galvanometer Scanner with Infrared Fringe Projection in High Speed. Appl. Sci..

[5] Petkovic T., Pribanic T., Donlic M., Sturm P. Efficient Separation Between Projected Patterns for Multiple Projector 3D People Scanning. Proceedings of the IEEE International Conference on Computer Vision Workshops.

[6] Holroyd M., Lawrence J. An analysis of using high-frequency sinusoidal illumination to measure the 3D shape of translucent objects. Proceedings of the CVPR 2011.

[7] Kobayashi T., Higo T., Yamasaki M., Kobayashi K., Katayama A. Accurate and Practical 3D Measurement for Translucent Objects by Dashed Lines and Complementary Gray Code Projection. Proceedings of the 2015 International Conference on 3D Vision.

[8] Chen S. (2022). Intraoral 3-D Measurement by Means of Group Coding Combined with Consistent Enhancement for Fringe Projection Pattern. IEEE Trans. Instrum. Meas..

[9] Yin W., Che Y., Li X., Li M., Hu Y., Feng S., Lam E.Y., Chen Q., Zuo C. (2024). Physics-informed deep learning for fringe pattern analysis. Opto-Electron. Adv..

[10] Zuo C., Qian J., Feng S., Yin W., Li Y., Fan P., Han J., Qian K., Chen Q. (2022). Deep learning in optical metrology: A review. Light Sci. Appl..

[11] Liu H., Yan N., Shao B., Yuan S., Zhang X. (2024). Deep learning in fringe projection: A review. Neurocomputing.

[12] Feng S.J., Chen Q., Gu G.H., Tao T.Y., Zhang L., Hu Y., Yin W., Zuo C. (2019). Fringe pattern analysis using deep learning. Adv. Photonics.

[13] Zhang L., Chen Q., Zuo C., Feng S. (2020). High-speed high dynamic range 3D shape measurement based on deep learning. Opt. Lasers Eng..

[14] Li Y.X., Qian J.M., Feng S.J., Chen Q., Zuo C. Single-shot spatial frequency multiplex fringe pattern for phase unwrapping using deep learning. Proceedings of the Optics Frontier Online 2020: Optics Imaging and Display.

[15] Chi W.L., Choo Y.H., Goh O.S. (2022). Review of Generative Adversarial Networks in Image Generation. J. Adv. Comput. Intell. Intell. Inform..

[16] Rajshekhar G., Rastogi P. (2012). Fringe analysis: Premise and perspectives. Opt. Lasers Eng..

[17] Su X.Y., Chen W.J. (2001). Fourier transform profilometry: A review. Opt. Lasers Eng..

[18] Kemao Q. (2007). Two-dimensional windowed Fourier transform for fringe pattern analysis: Principles, applications and implementations. Opt. Lasers Eng..

[19] Zhong J.G., Weng J.W. (2004). Spatial carrier-fringe pattern analysis by means of wavelet transform: Wavelet transform profilometry. Appl. Opt..

[20] Zuo C., Feng S., Huang L., Tao T., Yin W., Chen Q. (2018). Phase shifting algorithms for fringe projection profilometry: A review. Opt. Lasers Eng..

[21] Zhang S. (2018). Absolute phase retrieval methods for digital fringe projection profilometry: A review. Opt. Lasers Eng..

[22] Isola P., Zhu J.Y., Zhou T.H., Efros A.A. Image-to-Image Translation with Conditional Adversarial Networks. Proceedings of the 30th IEEE Conference on Computer Vision and Pattern Recognition (CVPR 2017).

[23] Zhou Z.W., Siddiquee M.M.R., Tajbakhsh N., Liang J.M. (2020). UNet plus plus: Redesigning Skip Connections to Exploit Multiscale Features in Image Segmentation. IEEE Trans. Med. Imaging.

[24] Liu J., Lin R., Wu G., Liu R., Luo Z., Fan X. (2023). CoCoNet: Coupled Contrastive Learning Network with Multi-level Feature Ensemble for Multi-modality Image Fusion. Int. J. Comput. Vis..

[25] Mirza M., Osindero S.J.C.S. (2014). Conditional Generative Adversarial Nets. arXiv.

[26] Popescu D., Deaconu M., Ichim L., Stamatescu G. Retinal Blood Vessel Segmentation Using Pix2Pix GAN. Proceedings of the 2021 29th Mediterranean Conference on Control and Automation (MED).

[27] Su Z., Zhang Y., Shi J., Zhang X.-P. (2023). A Survey of Single Image Rain Removal Based on Deep Learning. ACM Comput. Surv..

[28] Lai B.-Y., Chiang P.-J. (2023). Improved structured light system based on generative adversarial networks for highly-reflective surface measurement. Opt. Lasers Eng..

[29] Li Y.X., Qian J.M., Feng S.J., Chen Q., Zuo C. (2022). Deep-learning-enabled dual-frequency composite fringe projection profilometry for single-shot absolute 3D shape measurement. Opto-Electron. Adv..

[30] Yu H., Chen X., Huang R., Bai L., Zheng D., Han J. (2023). Untrained deep learning-based phase retrieval for fringe projection profilometry. Opt. Lasers Eng..

[31] Nguyen H., Novak E., Wang Z. (2022). Accurate 3D reconstruction via fringe-to-phase network. Measurement.

[32] Li Y.X., Qian J.M., Feng S.J., Chen Q., Zuo C. (2022). Composite fringe projection deep learning profilometry for single-shot absolute 3D shape measurement. Opt. Express.

[33] Fu Y., Huang Y., Xiao W., Li F., Li Y., Zuo P. (2024). Deep learning-based binocular composite color fringe projection profilometry for fast 3D measurements. Opt. Lasers Eng..

[34] Qian J., Feng S., Li Y., Tao T., Han J., Chen Q., Zuo C. (2020). Single-shot absolute 3D shape measurement with deep-learning-based color fringe projection profilometry. Opt. Lett..

[35] Jacob B., Kligys S., Chen B., Zhu M., Tang M., Howard A., Adam H., Kalenichenko D. Quantization and Training of Neural Networks for Efficient Integer-Arithmetic-Only Inference. Proceedings of the 2018 IEEE/CVF Conference on Computer Vision and Pattern Recognition.

[36] Xu S., Li Y.J., Liu C.J., Zhang B.C. (2024). Learning Accurate Low-bit Quantization towards Efficient Computational Imaging. Int. J. Comput. Vis..

