# Supplementary material for: Single Fringe Phase Retrieval for Translucent Object Measurements Using a Deep Convolutional Generative Adversarial Network
Source: Sensors (Basel). 2025 Mar 14;25(6):1823. doi: 10.3390/s25061823 (PMC11946306; doi:10.3390/s25061823)
Supplement: Supplementary file 1 [file sensors-25-01823-s001.zip › sensors-3491130-supplementary.pdf]

Supplementary Information

Table S1. Comparison Results of Absolute Phase of CA at Different Layers on Dataset 1.

CA Position			Dataset 1	
layer x^1	layer x^2	layer x^3	MAE	RMSE
			0.0751	0.4795
√			0.0759	0.4852
	√		0.0762	0.4876
		√	0.0773	0.4884
	√	√	0.0786	0.5042
√		√	0.0753	0.4825
√	√		0.0750	0.4828
√	√	√	0.0742	0.4785

In Table S1, the impact of incorporating the Channel Attention (CA) mechanism into different layers of the network is presented, showing varying effects on evaluation metrics. Notably, adding CA to a single layer increases error metrics, while its presence in multiple layers leads to different outcomes. Maximum error metrics are observed when CA is only in the second and third layers, whereas the lowest error metrics occur when CA is added to all three layers.

Speculatively, adding CA to only one layer's skip connection may over-optimize local features, neglecting global feature dependencies, leading to suboptimal information flow and disruption of feature representations in other layers. In contrast, adding CA to all layers' skip connections enables better capture of global feature dependencies, facilitating more effective information transmission and improving overall network performance. Furthermore, the network performs better when CA is added to lower-level features compared to higher-level features, possibly due to the richer local detail in lower-level features being more beneficial for the task at hand in this study.

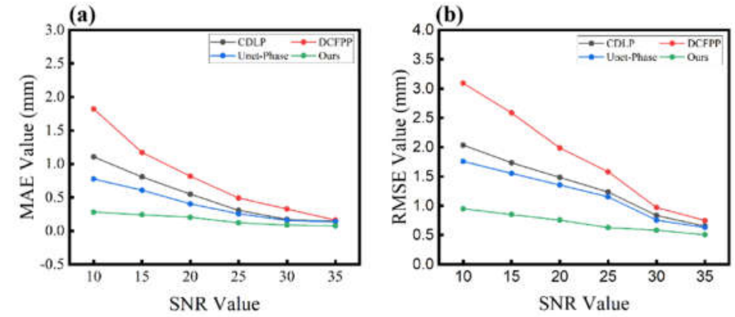

Figure S1. Evaluation metrics for datasets with different noise levels.

Gaussian white noise with signal-to-noise ratios (SNRs) of 10, 15, 20, 25, 30, and 35 was introduced into Dataset 1, generating multiple edge image datasets with varying noise levels. At an SNR of 10, the noise is pronounced, resulting in poor image quality, while at an SNR of 35, the noise is minimal, yielding high-quality images. The training process followed the same procedure as described in Sections 3.1 and 3.2. Figure 19 illustrates the variation of MAE/RMSE errors with re-spect to noise levels. It is evident that at lower SNRs, CDLP, Unet-Phase, and DCFPP exhibit significant instability, with notably high errors. As the SNR increases, their er-rors gradually decrease. In contrast, the green curve representing our method demon-strates a more stable trend compared to the other three curves, indicating superior ro-bustness across different noise levels. This stability underscores the effectiveness of our approach in handling noisy conditions.

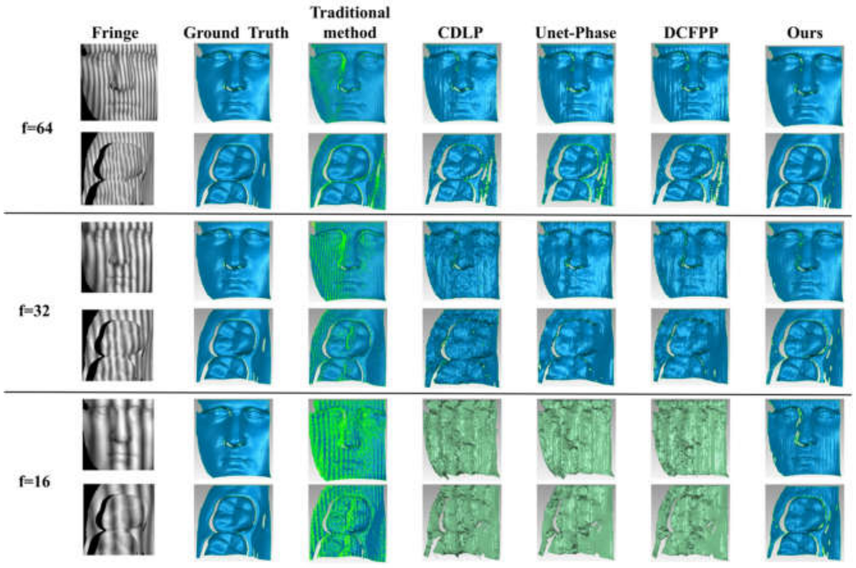

Figure S2. Reconstruction results of the four methods at different frequencies.

Figure S2 illustrates the reconstruction results of the four deep learning methods across these frequencies. The first column depicts the fringe patterns for each frequency, followed by reconstruction results from traditional method, CDLP, Unet-Phase, DCFPP, and Ours, respectively. The last column displays the ground truth obtained through the 12-step phase shifting method.

When the frequency is 64, all four networks yielded comparatively complete reconstruction results. However, CDLP, Unet-Phase, and DCFPP exhibited varying degrees of ripples, while our network maintained detailed reconstruction results. When the frequency is 32, the reconstruction accuracy of all networks declines due to the reduction in the number of fringes and the consequent decrease in feature information, leading to diminished accuracy in predicted phase information. The details in the reconstructions from CDLP, Unet-Phase, and DCFPP became blurred, with CDLP particularly suffering the most. Our method still achieved a relatively complete reconstruction but with some "vertical bars". When f is 16, the fringe map captured by the camera contained only four fringes, significantly reducing the feature information available to the networks. As a result, the reconstruction outputs from CDLP, Unet-Phase, and DCFPP were notably degraded, appearing chaotic, leading to an inability for the point cloud to be processed by Geomagic Wrap software and displayed in green. Despite reduced accuracy, our method still managed to produce a clear outline from an overall perspective. In summary, the proposed network demonstrated superior generalization performance compared to the other three networks.

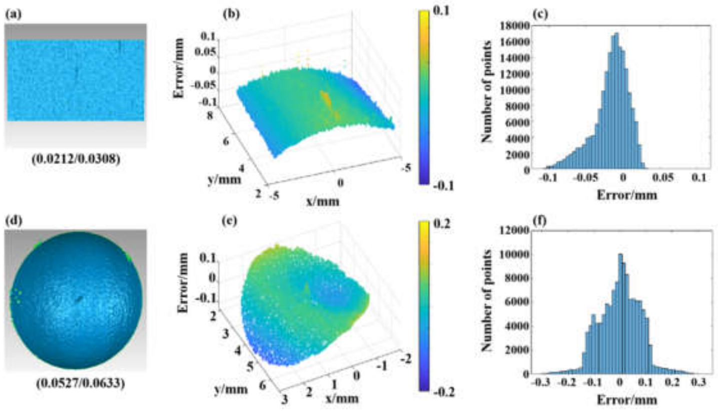

Figure S3. Precision analysis of standard ceramic plate and a standard ceramic sphere. (a, d) 3D reconstruction results by the proposed GAN-PhaseNet. (b, e) Error distribution. (c, f) RMS error.

Figure S3(a) and S3(d) display the reconstruction maps obtained through our method, while Figure S3(c) and S3(f) illustrate the error distributions for the plate and the standard ceramic sphere, respectively. The ground truth values were derived by fitting a plane or sphere to the 3D reconstruction data, resulting in MAE and RMSE of 0.0212/0.0308 for the plate and 0.0527/0.0633 for the sphere.
